# MicroRNAs as Potential Regulators of Glutathione Peroxidases Expression and Their Role in Obesity and Related Pathologies

**DOI:** 10.3390/ijms19041199

**Published:** 2018-04-14

**Authors:** Petra Matoušková, Barbora Hanousková, Lenka Skálová

**Affiliations:** Faculty of Pharmacy, Department of Biochemical Sciences, Charles University, 500 05, Hradec Králové, Czech Republic; hanouskb@faf.cuni.cz (B.H.); skaloval@faf.cuni.cz (L.S.)

**Keywords:** glutathione peroxidase, obesity, microRNA, bioinformatics

## Abstract

Glutathione peroxidases (GPxs) belong to the eight-member family of phylogenetically related enzymes with different cellular localization, but distinct antioxidant function. Several GPxs are important selenoproteins. Dysregulated GPx expression is connected with severe pathologies, including obesity and diabetes. We performed a comprehensive bioinformatic analysis using the programs miRDB, miRanda, TargetScan, and Diana in the search for hypothetical microRNAs targeting 3′untranslated regions (3´UTR) of GPxs. We cross-referenced the literature for possible intersections between our results and available reports on identified microRNAs, with a special focus on the microRNAs related to oxidative stress, obesity, and related pathologies. We identified many microRNAs with an association with oxidative stress and obesity as putative regulators of GPxs. In particular, miR-185-5p was predicted by a larger number of programs to target six GPxs and thus could play the role as their master regulator. This microRNA was altered by selenium deficiency and can play a role as a feedback control of selenoproteins’ expression. Through the bioinformatics analysis we revealed the potential connection of microRNAs, GPxs, obesity, and other redox imbalance related diseases.

## 1. Introduction

Obesity, defined as excessive fat accumulation, associated with increased risk of chronic diseases such as metabolic syndrome, diabetes mellitus, and cardiovascular disease is increasing worldwide. In general, the elucidation of the molecular mechanisms involved in promoting obesity and adipogenesis is of utmost importance [1]. To date, various platforms for identification of novel biomarkers have been employed. However, methodological limitations and biological variability impede the establishment of particular biomarkers [2]. Reactive oxygen species (ROS), such as hydrogen peroxide hydroxyl radical, and superoxide radical, are involved in the etiology of a wide range of diseases, including obesity and obesity-related pathologies. Excess cellular ROS accumulation has been shown to induce DNA damage, from point mutations to chromosomal aberrations. In addition to genotoxic effects, ROS activate signal transduction pathways, leading to changes in expression of many important genes. The cellular antioxidant system, which maintains the cellular balance of ROS production and removal, is composed of non-enzymatic compounds with redox activity (e.g., glutathione, vitamin E, and thioredoxin) as well as enzymes (e.g., superoxide dismutase, catalase, glutathione reductases, and glutathione peroxidases) [3,4].

Glutathione peroxidases (GPxs), a family of phylogenetically related enzymes, can be divided into three evolutionary groups arising from one Cystein (Cys)-containing ancestor: GPx1/GPx2, GPx3/GPx5/GPx6, and GPx4/GPx7/GPx8. Mammalian GPx1–4 are selenoproteins with selenocysteine (Sec) in their catalytic center. They appear to possess an antioxidant function at different locations and cellular compartments. GPx5, GPx7, and GPx8 contain Cys instead of Sec in the active center. GPx6 is a selenoprotein in humans but not in rodents. GPx1, 2, 3, 5, and 6 are homotetramers, while the others are monomers [5].

GPxs catalyze the reduction of H_2_O_2_ or organic hydroperoxides forming water and the corresponding alcohols, respectively, mainly using glutathione (GSH) as a reductant. With the increasing evidence that hydroperoxides are not simply harmful ROS but mediators of physiological processes [6], the role of GPxs also needs to be re-viewed [5]. Hydroxyperoxides that can play a specific role in the redox signaling must be sensed by proteins with highly reactive thiol or selenol groups which transfer the message. GPxs may act as such sensors. Furthermore, GPxs can certainly regulate the concentration of hydroperoxides and in this way affect the respective pathways [7]. The significance of GPxs can be deduced from the observation that knockout of some GPxs has fatal consequences, and that dysregulated GPx expression is connected with severe pathologies, including obesity and diabetes [8,9].

The relationship between GPxs and obesity has been observed in animal models as well as in human studies. The changed expression/activities of GPxs were found in the adipose tissues of obese individuals [10]. GPx1 is considered to play important role in obesity, insulin resistance, and atherosclerosis. Specifically, the action of intracellular ROS required for insulin sensitizing may be impaired by increased GPx1 activity resulting in progression of insulin resistance. [11]. GPx3 activity and expression has been shown to be decreased in plasma of obese individuals, with its expression being restored in mice by treatment with antioxidants or rosiglitazone [12]. GPx7 loss resulted in enhanced oxidative stress and increased adipose tissue mass in mice [10]. Certain single nucleotide polymorphisms for *GPx4–6* genes were found to be associated with prepubertal childhood obesity in Spain [13]. Taking together, the importance of GPx in obesity and related pathologies is evident. For this reason, findings regarding regulatory mechanisms that control GPxs expression are of a great importance.

In addition to other molecules, microRNAs (miRNAs) could play a significant role in the regulation of GPxs expression. miRNAs, small single-stranded RNA molecules, consist of about 22 nucleotides which imperfectly hybridize mostly to 3′UTR of a gene transcript, typically leading to translation inhibition, lower protein expression, and/or transcript degradation [14]. miRNAs exhibit unique expression patterns in specific tissues, with the aberrant expression of miRNAs associated with cellular dysfunction and diseases [15,16]. Variations in miRNA levels can occur as a result of single nucleotide polymorphisms, epigenetic modifications, developmental changes or the progression of tissue from a healthy to diseased state.

To date miRNAs have been shown to play a vital role in almost every cellular process, ranging from differentiation and development to cell death [14]. A variety of metabolic processes, studied in the context of diabetes and obesity are as well affected by miRNAs [17]. It is not surprising that miRNAs have been also found to control stress responses including redox balance [3]. miRNAs called “redoximiRs” are capable of reducing ROS generation and enhancing endogenous antioxidant defenses [18]. However, concerning GPxs, part of the cellular antioxidant defense system, only a few pieces of evidence have been made available as of yet.

## 2. Bioinformatic Analysis: MicroRNAs and GPxs

In the present study, we used a bioinformatics approach to determine probable miRNAs that interact with the 3′UTR of individual GPx genes. We attempted to create a list of in silico-identified miRNAs predicted to regulate GPxs 1–8. Using literature searches, we cross-referenced these miRNAs and their links to oxidative stress, obesity, and related pathologies. Furthermore, in each section, we first discuss the literature dealing with respective GPx containing observed correlations or functional analysis conducted, which were obtained from miRWalk tool. Furthermore, we reviewed the reports of in silico identified miRNAs.

**Figure 1:**
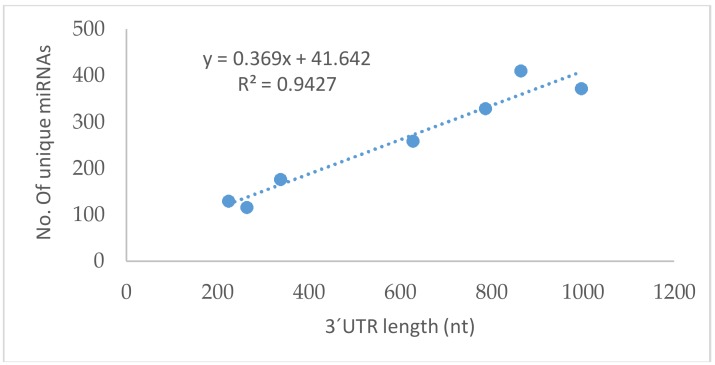
Correlation between 3′UTR length (nt) of selected genes and the number of unique miRNAs predicted by all used algorithms (*GPx8* is not included in the analysis.)

**Figure 2:**
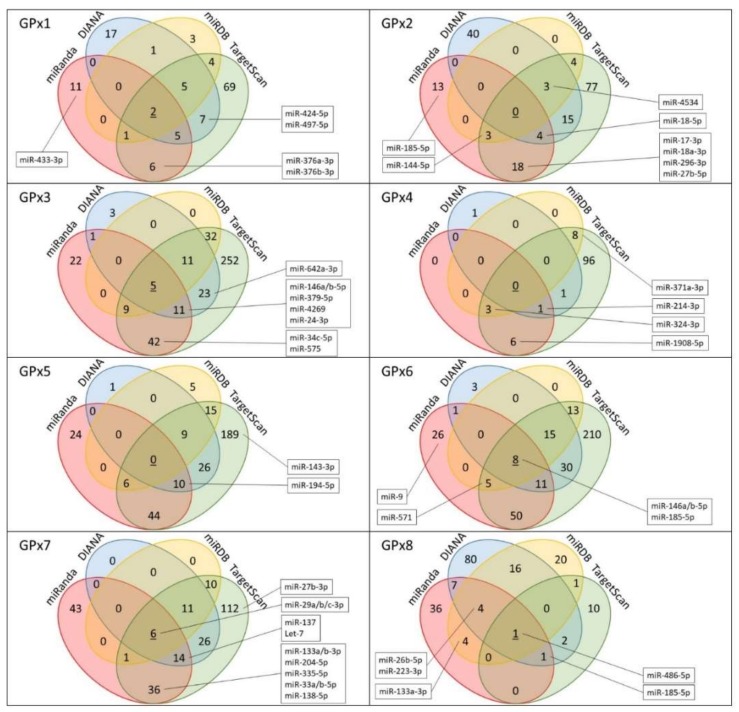
The Venn diagrams representing the number of microRNAs predicted to target GPxs identified by each program used. Identified microRNAs in boxes have reported connection to oxidative stress and obesity.

We used four open-access online programs (TargetScan, DIANA, miRanda, and miRDB) to identify putative miRNAs targeting GPxs (Supplementary materials). The four programs use different algorithms of varying strengths based on the optimal alignment between the miRNA seed sequence and sequence conservation in the miRNA recognition element region. We found that all GPxs could be predicted to be regulated by miRNAs (Table 1 and Table S1). A significant correlation was observed between the total number of unique miRNAs predicted by all programs and the length of 3′UTR of each *GPxs* (Figure 1). *GPx8* has not been included in this analysis. Due to the very long 3′UTR (4869 nt) of *GPx8*, we included only conserved sites from the TargetScan prediction, which significantly lowered the number of predicted miRNAs. The programs intersections were carefully analyzed (Figure 2). Several miRNAs were predicted by all four programs for some of the *GPxs*, highly suggesting that they are a true regulator of respective GPx, which, however, can be verified only experimentally.

### 2.1. GPx1

GPx1, the first selenoprotein identified, is mainly known as an enzyme which acts against oxidative stress. GPx1 is a homotetramer ubiquitous in the cytosol and mitochondria, highly expressed in erythrocytes and in cells including vascular endothelium. The catalytic site of the enzyme contains the amino acids Sec, Lys, and four Arg, which provides GPx1 with the highest specificity for GSH out of all GPxs. GPx1 reacts mainly with H_2_O_2_, hydroperoxy lysophosphatides, and soluble low molecular mass hydroperoxides (e.g., *t*-butyl hydroperoxide, cumene hydroperoxide), as well as hydroperoxy fatty acids [5]. In the brain mitochondria, no GSH is present and therefore GPx1 uses γ-glutamylcysteine as a reductant for H_2_O_2_ removal [19].

Changes in the expression of GPx1 may have various consequences. The overexpression of GPx1 was shown to protect mice from acute oxidative stress, however, later hyperglycemia, hyperinsulinemia, insulin resistance, obesity, as well as increased β-cell mass developed. The lack of H_2_O_2_ caused by overexpressed GPx1 can inhibit insulin signaling and cause the overproduction of insulin. Incidences of hyperinsulinemia caused by GPx1-overexpression were not diminished by dietary limitation [11]. Conversely, a deficiency of GPx1 may cause endothelial dysfunction, atherosclerosis, abnormal structural changes in vasculature and myocardium, coronary heart disease, and heart failure. The *GPx1* gene contains a polymorphism at codon 198, which leads to a Pro198Leu substitution, a change associated with morbid obesity [10], coronary artery disease, intracerebral hemorrhage, diabetic associated atherosclerosis, and increased carotid intima-media thickness as well as a prevalence of cardiovascular and peripheral vascular disease in patients with type 2 diabetes mellitus [20].

Because GPx1 is major antioxidant enzyme catalyzing the breakdown of H_2_O_2_, it is not surprising that Wang et al. [21] reported a significant increase in GPx1 expression in cardiomyocytes after H_2_O_2_ treatment. Two miRNAs were experimentally verified to regulate GPx1. The inverse correlation with miR-181a was observed and subsequently the regulation of GPx1 was functionally confirmed [21]. Antioxidant response in human endothelial cells was disturbed by oscillating glucose accompanied by miR-185-5p induction. Furthermore, knockdown of miR-185-5p caused a significant upregulation of GPx1 [22]. However, of note is that, of the programs we used, none identified miR-181a or miR-185-5p as a putative regulator of GPx1. In analyzing *GPx1*, TargetScan offers two 3′UTR transcripts with different lengths to explore. Only the longer (816 nt) transcript contains target sites for both mentioned miRNAs. Nevertheless, we focused on the shorter transcript (222 nt), as its length corresponds to the 3′UTR length of the *GPx1* gene in the GenBank (NM_000581), the very same sequence that La Sala also refers to [22]. Correspondingly, other programs we used consider the shorter version of 3′UTR as well.

Many unique miRNAs with possible interactions with the 3′UTR of *GPx1* genes have been predicted (Table 1 and Table S1). Among miRNAs identified by more than one program, there are several miRNAs with known relationships to obesity, diabetes mellitus, and/or oxidative stress. The serum miR-376a-3p was significantly diminished in obese serum and could serve as a potential predictive tool for distinguishing obese patients from normal healthy controls [17]. miR-376a-3p was associated with the senescence of human umbilical vein endothelial cells (HUVEC) [23]. The increased expression of miR-376b-3p attenuated starvation-induced autophagy in cell lines [24]. Three miRNAs, including miR-376b-3p, were down-regulated in obese women in a weight-loss trial in which they experienced no weight loss (non-responders) in comparison to successful responders (weight loss >5%) [25].

In neuronal cultures, miR-424-5p treatment decreased H_2_O_2_-induced neuronal apoptosis, reduced ROS and malondialdehyde levels, and affected the redox-sensitive transcription factor nuclear factor erythroid 2-related factor 2 (Nrf2), a central regulator of cellular response to oxidative stress [26]. Over-expression of mmu-miR-322, orthologous to human miR-424, protected hearts in mice from the effects of a high-fat diet. The transcriptome analysis revealed this cardio-protection could be associated with an enrichment of genes involved in mitochondrial function and fatty acid oxidation. These results indicate miR-424-5p as a potential therapeutic target with an emphasis on cardiovascular disease [27].

The insulin receptor is the target gene of miR-497-5p, and elevated miR-497-5p might induce hepatic insulin resistance in rats with high-fat diet through the inhibition of the expression of the insulin receptor [28]. In another study, miR-497-5p inhibited the inflammation and apoptosis of a spinal cord ischemia-reperfusion injury [29]. miR-497-5p was increasingly up-regulated when atherosclerosis progressed in apolipoprotein E (ApoE)-deficient mice [30] as well as during the course of aging of rats which correlated with liver cells senescence [31].

Among dozens of miRNAs with a possible *GPx1* interaction predicted only by one program (Table S1), one additional miRNA, miR-433, is a redoximiR. RedoximiRs are miRNAs reported in connection to oxidative stress. The miR-433 overexpression in HUVEC cells showed a downregulation of glutathione synthesis and resulted in reduced antioxidant and redox potentials [32].

### 2.2. GPx2

GPx2 is homotetramer with the amino acid Sec in the catalytic center. It is expressed mainly in the epithelium of the gastrointestinal (GI) system (sometimes referred to as GI-GPx), the esophagus, and in humans also in the liver. *GPx2* is an Nrf2-target gene, therefore it can be induced by antioxidants [33]. In the epithelium of the gastrointestinal system, the highest concentration was found at the crypt bases and in Paneth cells, where GPx2 may protect against the absorption of food-born hydroperoxides and contribute to immune response [34]. A possible anti-inflammatory function was ascribed to GPx2, as GPx2 knockout led to an increase in cyclooxygenase 2 expression and prostaglandin E2 production in HT29 carcinoma cells [35]. GPx2 also plays an important role in embryogenesis, specifically in organogenesis. A high concentration was found in rapidly growing tissues and in extraembryonic tissues, where GPx2 protected the embryo from ROS [36].

Selenium is incorporated into selenoproteins as the amino acid Sec. A deficiency in Se intake alters the levels and activity of selenoproteins. A hierarchy of selenoproteins in which some are more sensitive to changes in Se intake than others has been proposed [37]. GPx2 scores very high in this hierarchy, as proved with the Se suboptimal supplementation of a Caco-2 cell line [38].

The regulation of GPx2 expression has been experimentally proven for two miRNAs: miR-17-3p and miR-185-5p. miR-17-3p is expressed from an miR-17-92 cluster; however it is less expressed (commonly regarded as a passenger strand) than miRNA from the other pre-miRNA hairpin arm, miR-17-5p, which is involved in the pathogenesis of cancer and diabetes. miR-17-5p was significantly elevated in the group of obese patients in comparison to normal ones [39]. The study of Xu et al. [40] demonstrates that miR-17-3p is able to suppress GPx2 and other important antioxidant enzymes such as manganese superoxide dismutase (MnSOD) and thioredoxin reductase-2 (TrxR2) in prostate cancer PC-3 cells. The association between the second miRNA, miR-185-5p, was deduced from correlations in silencing experiments in intestinal cell lines; when the miR-185-5p is silenced the expression of GPx2 rises [38]. miR-185-5p involvement in oxidative stress, obesity, and diabetes has been shown in many studies. Oxidative stress regulates the expression of miR-185-5p, a miRNA affecting calcium cytosolic concentration [41]. The concentration of miR-185-5p in plasma increased during the progression of type 2 diabetes [42]. In accordance, miR-185-5p was shown to play an important role in the regulation of insulin secretion and β-cell growth in diabetes [43]. Oxidative stress-associated cell death through enhanced DNA damage is caused by hyperoxia-induced miR-185-5p in lung epithelial cells [44]. Moreover, miR-185-5p was identified as a regulator of de novo cholesterol biosynthesis and low density lipoprotein uptake [45] or lipid metabolism in mice with non-alcoholic fatty liver disease [46]. On the other hand, the inhibition of miR-185-5p was found to reduce plasma LDL levels and atherosclerotic plaque size in ApoE-deficient mice [47]. miR-185 was downregulated in aging rat livers [31].

Surprisingly, both miRNAs from pre-mir-18a, i.e., miR-18a-3p and miR-18a-5p, which are expressed from a miR-17-92 cluster, were predicted to regulate GPx2 expression. miR-18a-5p is one of several miRNAs with increased expression after the consumption of long-chain polyunsaturated fatty acids; this miRNA is connected with an improvement of metabolic syndrome markers [48]. Interestingly, miR-144-5p, identified by three programs, is the other strand of commonly expressed miR-144-3p, a miRNA which is involved in direct regulation of Nrf2 [49].

The specific downregulation of miR-296-3p coupled to upregulation of its targets, such as insulin-like growth factor 1 receptor beta and tumor necrosis factor alpha (TNFα), is a major determinant of mammalian pancreatic alpha cell resistance to apoptosis induction by cytokines [50]. The study of miRNAs abundance in the serum samples from people with type 2 diabetes mellitus (T2DM) as compared to that of healthy individuals revealed five significantly different miRNAs, including miR-4534 [51].

Besides miR-144-5p, among redoximiRs, miR-27b-5p was identified as a GPx2 regulator. miR-27b-5p is an oxidative-stress responsive miRNA that was found to modulate the nuclear factor-κB (NFκB) pathway as well as the overexpression of the miR-27b-5p suppressed lipopolysaccharide-induction of NF-κB [52]. The level of circulating miR-27a-5p has been found to be significantly higher in obese children [53].

### 2.3. GPx3

GPx3, the major antioxidant enzyme in plasma, is a homotetramer containing Sec in the catalytic center and is actively secreted into the plasma as a glycosylated protein [5]. GPx3 is also associated with the basement membranes of epithelial cells in the intestine, epididymis, bronchi, and type II pneumocytes and it is expressed in various other tissues, such as the proximal convoluted tubule of the kidney, as well as in thyroid colloid lumen and adipose tissue [54]. The function of GPx3 is to scavenge H_2_O_2_ and lipoperoxides in the plasma to reduce systematic oxidative stress and to maintain the bioavailability of vascular nitric oxide.

As obesity is linked to oxidative stress and inflammation, the involvement of Gpx3 has been studied several times, however contradictory results have been reported. Although white and brown adipose tissue express high amounts of GPx3, this expression was reduced in obese mice and GPx3 protein levels, with total GPx3 plasma activity also reduced in obese human subjects [12]. On the contrary, one study from central Mexico reported that patients with overweight or obesity had increased serum levels of GPx3 [55].

miR-92a-3p has repeatedly been reported in connection to oxidative stress, brown fat activity, and endothelial ageing. This miRNA was identified as validated miRNA by crosslinking, ligation, and sequencing of hybrids (CLASH) to regulate GPx3 expression. Since then no program identified this miRNA as putative regulator of GPx3; a noncanonical binding was proposed. However, miR-92a-5p, the other strand of miR-92a duplex, was identified as putative regulator by three programs used, which opens up the possibility of fine regulation by this miRNA and action of both strands.

Two inflammation-related miRNAs from the miR-146 family, miR-146a-5p and miR-146b-5p, were identified as putative regulators of GPx3. Upon inflammation in human adipocytes, miR-146a-5p was reported as the most upregulated miRNA [56]. Previously associated with atherosclerotic disease and diabetes, miR-146a-5p was suggested as a potential therapeutic target for the treatment of diabetic retinopathy [57]. miR-146a-5p gene polymorphism was related to the increased risk of atherosclerosis in diabetic patients [58]. Furthermore, the antioxidant ubiquinol-10 may fine tune the inflammatory response via the moderate reduction of miR-146a-5p expression [59]. miR-146b-5p is an intergenic miRNA highly expressed in mature adipocytes, while very little expressed in human mesenchymal stem cells and human visceral preadipocytes. miR-146b-5p was able to inhibit the proliferation of visceral preadipocytes and promote their differentiation [60]. The up-regulation of miR-146b-5p was also observed during the development of obesity in mice fed a high-fat diet [1]. miR-146b-5p was downregulated in monocytes of obese persons, and it seems to be a major mediator of the anti-inflammatory action of globular adiponectin, a well-recognized antidiabetic adipokine. Low globular adiponectin decreased miR-146b-5p in monocytes and increased mitochondrial ROS [61]. Moreover, miR-146b-5p can regulate the inflammatory response by attenuating cytokine signaling via the NFκB pathway. The mature human adipocytes responded to proinflammatory cytokines (TNFα and interleukin-6) by highly increasing the expression of miR-146b-5p [62]. Furthermore, Li et al. [63] reported that the inhibition of miR-146b-5p led to increased hypoxia-induced apoptosis and that this had a protective role in cardiac chronic hypoxia [63].

Many other miRNAs have been identified as possible GPx3 regulators reported in connection to oxidative stress and related pathologies. Examples include miR-34c-5p, reported as induced by hypoxia in mouse lungs [64] and significantly over-expressed in T2DM monocytes [65]; miR-379-5p, upregulated in mice fed with a high-fat diet [1]; miR-575, expressed in the amnion of obese pregnant woman only, whereas not detected in the amnion of controls [66]; miR-642a-3p, an adipocyte-specific miRNA upregulated in the fat depots of obese subjects [67]; an exosomal miR-4269 downregulated in obese donors [68]; miR-24-3p, which promotes adipocyte differentiation by directly targeting mitogen-activated protein kinase 7 (MAPK7) signaling [69] and with a high fat diet shows upregulated expression in mice livers, with the miR-24 inhibitor suggested as potential therapeutic agent for nonalcoholic fatty liver disease [70].

### 2.4. GPx4

The structurally monomeric GPx4 is a selenoprotein initially characterized as a lipid peroxidation inhibiting protein, since GPx4 protects membrane lipids against oxidation and reduces various forms of hydroperoxides even if they are incorporated into biomembranes or lipoproteins [5]. GPx4 has three different isoforms derived from the same gene: the cytosolic (cGPx4), mitochondrial (mGPx4), and sperm nuclear (snGPx4) isoforms. While cGPx4 is ubiquitous in cells, mGPx4 and snGPx4 are mainly expressed in the testis and only marginally in other tissues [3]. Systemic knockout of the entire *GPx4* gene in mice had lethal effects, while mGPx4 knockout female mice developed normally and were completely viable, but male mice were infertile and their sperm showed structural abnormalities [71].

Variants of GPx4 resulting in diminished activity have been associated with obesity inflammation and cardiovascular diseases [13]. In a model of GPx4 haplo-insufficiency, a causal role causing significantly increased lipid peroxide-derived aldehydes was demonstrated and presented in obesity as well as in more severe cardio-metabolic diseases including dyslipidemia, glucose intolerance, liver steatosis, cardiac fibrosis, and hypertrophy. Katunga et al. [72] proposed a model of GPx4 as a protective adaptation to obesity. Obesity/nutrient overload is linked to permanent oxidative stress and increased peroxidation is followed by GPx4 compensatory upregulation [72].

Identified as a putative regulator of GPx4 by three programs, miR-214-3p has been previously associated with increased oxidative stress in old mice livers as well as age-related oxidative defense decline by targeting various classes of glutathione *S*-transferases [73]. Furthermore, miR-214-3p has been reported to protect erythroid cells against oxidative stress through Nrf2. miR-214-3p expression was transcriptionally repressed by Nrf2, which enabled a release of the repression of mir-214-3p targets (e.g., ATF4—A key transcriptional factor involved in cytoprotection upon stress) [74]. Such a regulation might well correspond to a possible protective role by the released repression of GPx4. miR-214-3p also down-regulates glutathione reductase and cytochrome P450 oxidoreductase promoting alcohol-induced oxidative stress in liver cells [75]. miR-324-3p was among decreased circulating miRNAs downregulated in gestational obesity, and is also associated with pregnancy weight gain [76] as well as was previously reported as an inducer of the pro-inflammatory transcription factor NFκB [77]. Another identified miRNA, miR-371a-3p (expressed from cluster 371–373) has been reported to be associated with drug tolerance and oxidative stress management through targeting peroxiredoxin 6 [78]. In addition, miR-371a-3p was previously identified as a senescence-associated miRNA upregulated in the long-term culture of mesenchymal stromal cells and as a positive adipogenic regulator [79]. miR-1908-5p is highly expressed in mature adipocytes; stimulation with free fatty acids resulted in downregulation of this miRNA, which indicates its involvement in the regulation of obesity development [80]. Finally, the overexpression of miR-1908-5p inhibited differentiation and promoted proliferation of human multipotent adipose-derived stem cells [81].

### 2.5. GPx5

GPx5 is homotetrameric, epididymal specific, selenium-independent enzyme containing Cys in its active center and together with GPx3 they account for more than 95% of epididymal GPx content, with their expression controlled by testicular factors, epididymis-specific transcription factors, and androgens [82]. GPx5 is released from epithelial cells into the epididymal lumen, where it binds to the head of spermatozoa. A study from 2013 showed an important role of GPx5 in the maintenance of cells and DNA integrity. The severe reduction in GPx5 participates in the vulnerability of human sperm to oxidative stress [83]. In a mammalian cell line expressing recombinant GPx5, increased resistance of cells to oxidative challenge and decreased levels of lipid peroxidation was shown [83]. In general, GPx5 protects sperm from peroxide-mediated attacks.

In addition to the important role of GPx5 in human fertility, an association of the single nucleotide polymorphism (SNP) rs445870 within *GPx5* with higher childhood obesity was found, with a minor allele of this genetic variant related to an increase in body mass index (BMI) [13].

Among the 330 unique miRNAs with predicted binding to 3′UTR of *GPx5* gene, according to the miRNA expression atlas (http://www.microrna.org/microrna/getExprForTissues.do), only one miRNA, miR-143-3p, is significantly expressed in epididymis. This miRNA has a role in adipogenic differentiation and is also closely related to obesity [84]. However, the expression of miR-143-3p is not essential for differentiation, as demonstrated by systematic silencing experiments in mice [85]. miR-143-3p level was lower in obese human subjects in comparison to controls [86]. Another predicted miRNA with proved (but low) expression in epididymis is miR-194-5p. The expression of miR-194-5p was significantly reduced in humans with pre-diabetes and established diabetes in comparison to healthy individuals. Knockdown of miR-194-5p in L6 skeletal muscle cells led to an increase in basal and insulin-stimulated glucose uptake and glycogen synthesis [87]. In diabetic mice, miR-194-5p expression was also down-regulated [88]. The down-regulation of miR-194-5p is likely an adaptive response to augment tissue glucose uptake and metabolism in the face of insulin resistance in patients with diabetes [87]. In the adult liver, miR-194-5p is a target of transcription factor hepatocyte nuclear factor 1-alpha [89]. A high fat maternal diet during pregnancy and lactation altered hepatic expression of the insulin like growth factor-2 and of some miRNAs, including miR-194-5p in the adult offspring [90].

### 2.6. GPx6

GPx6, a close homologue to GPx3, is a homotetrameric selenoprotein in humans but a CysGPx in rodents and other species. Up to the present date, information about GPx6 remains very limited. The mRNA of *GPx6* is expressed in the olfactory epithelium and is involved in the metabolism of odorants and the olfactory system in mammals. Together with other *GPx* genes, *GPx6* is upregulated in the cochleae in mice with age-related hearing loss [91]. GPx6 protein levels were altered in the striatum of Huntington’s disease patients and GPx6 interacts genetically with mutant huntingtin [92]. In addition, the association of SNP rs406113 of *GPx6* gene was linked to a higher childhood obesity risk, with the minor allele of this genetic variant related to an increase in BMI [13].

In GPx6 regulation, the participation of 40 miRNAs has been predicted by at least three software programs. Several of these are clearly connected to obesity and obesity related pathologies.

Among the predicted *GPx6*-interacting miRNAs, miR-185-5p regulates GPx1 and GPx2 and along with miR-146a/b-5p was predicted as a GPx3 regulator. For this reason, the role of these miRNAs in obesity and related pathologies is discussed in previous sections of the present article. The up-regulation of miR-539-5p was shown to be involved in the inhibition of de novo lipogenesis induced by resveratrol in white adipose tissue [93]. Five miRNAs, including miR-571, were significantly upregulated in T2DM patients in comparison to healthy individuals [94]. miR-9, a member of the redoximiRs group, was significantly differentially expressed, with 10-fold changes in subcutaneous adipose tissue between lean and obese pigs [95]. In addition, miR-9 is speculated to be involved in insulin secretion [96].

Interestingly, a number of predicted miRNAs that may bind to GPx6 were shown in both arms of the precursor miRNA (Table S1). For example, oxidative stress related miR-205-5p and -3p were both predicted by two programs. One tumor suppressive miRNA, miR-205-5p, was increased after ROS induction and high cytosolic calcium levels [41].

### 2.7. GPx7

GPx7 is monomeric enzyme containing Cys instead of Sec in its catalytic center. It was initially described as a cytosolic protein, but was later found in the lumen of endoplasmic reticulum (ER). Due to its homology to phospholipid hydroperoxide GPx (PHGPx = GPx4), GPx7 is also referred to as the non-selenocysteine containing phospholipid hydroperoxide glutathione peroxidase NPHGPx [97]. Even if GPx7 contains the GSH-binding domain, unlike other GPxs, it shows no GPx activity and reacts mainly with thioredoxin. GPx7 reduces the accumulation of ROS in cells, and is significantly involved in the maintenance of redox homeostasis as well as participating in the correct protein folding as an intracellular stress sensor/transmitter to transfer the signal to its interacting proteins [98].

GPx7 is highly expressed in preadipocytes in white adipose tissue, but not in mature adipocytes. A lower expression was also observed in macrophages and endothelial cells. Generally, GPx7 deficiency did not lead to embryonic lethality in mice. These mice carried multiple abnormalities such as glomerulonephritis, splenomegaly, cardiomegaly, vasculitis, and fatty liver, all of which are associated with numerous diseases [99,100]. A deficiency of GPx7 facilitated preadipocytes to differentiate to adipocytes and promoted both adipocyte hypertrophy and hyperplasia through the ROS dependent activation of the protein kinase A/CAAT box/enhancer-binding protein β (PKA/C/EBPβ) signaling pathway. The SNPs found near the human *GPx7* gene correlated with lower GPx7 expression in abdominal fat and white adipose tissue, higher plasma malondialdehyde levels, weight gain, obesity, and higher BMI. The results of many studies showed a protective role of GPx7 against fat accumulation and adipogenesis in mice and human by targeting redox homeostasis [101].

It is no surprise that many of the identified putative miRNAs as GPx7 regulators have shown an association to obesity and diabetes. One of the two conserved miRNAs identified by TargetScan as a potential regulator of GPx7 was miR-137, which controls the proliferation and differentiation of human adipose tissue stromal cells [102]. The down-regulation of miR-137 improved high glucose-induced oxidative stress through AMP-activated PKA1 [103] as well as H_2_O_2_-induced cardiomyocyte apoptosis through a cell division cycle 42 homolog [104]. Another identified conserved miRNA was the miR-29 family, the three members a, b, and c of which share a seed sequence and are all implicated in diabetes, obesity, and cardiovascular diseases regulating glucose homeostasis [105,106,107]. Among all of the reported activities, here are some examples; miR-29b-3p promoted the adipogenic differentiation through the transcription factor Sp1 (specificity protein 1)-mediated inhibition of TNFα [108], miR-29c-3p promoted senescence of human mesenchymal cells [109], and the up-regulation of miR-29a-3p inhibited p85 alpha subunit of phosphoiositide-3-kinase in insulin signaling cascade [110]. Many members of the let-7 miRNA family (including miR-98) were identified as putative regulators of GPx7 by all of the four programs used. The redoximiRs of the let-7 family are involved in glucose homeostasis and insulin sensitivity, and are as well broadly associated with obesity [111]. A recent meta-analysis of adipocyte differentiation and obesity studies revealed the great importance of let-7 family in adipogenesis [112]. On the other hand, the overexpression of let-7b or let-7c initiated the downregulation of Bach1, a transcriptional repressor of heme oxygenase 1, a finding which suggests the protection of human hepatocytes from oxidant injury [113]. Two programs predicted miR-133a/b-3p and miR-204-5p as ranking among the differentially expressed miRNAs found in association with a long-term high fat diet [1]. miR-133-3p is involved in brown adipocyte regeneration and was proposed as an important therapeutic target for the treatment of obesity [114]. miR-204-5p promoted the adipogenic differentiation of mesenchymal cells and inhibited the activation of the Wnt/β-catenin signaling pathway [115]. miR-204-5p is induced in diabetes and is involved in β-cell function [116]. Two programs predicted miR-335-5p as a putative regulator of GPx7. This miRNA is involved in adipogenesis and is related to inflammation [117]. Furthermore, miR-335-5p is upregulated in the adipose tissue of genetically and high-fat diet-induced obese mice [118,119]. Only two programs identified miR-33a/b-5p, but TargetScan predicted two binding sites for this miRNA in GPx7 3′UTR. miR-33, an intronic miRNA transcribed from sterol regulatory element-binding protein, is a transcription factor involved in cholesterol, fatty acids, and phospholipid synthesis. The involvement of miR-33a-5p in lipid homeostasis was repeatedly reported [120,121,122,123]. The therapeutic silencing of miR-33-5p in a mouse model of diet-induced obesity promoted the notion of whole-body oxidative metabolism [124]. miRNAs from both arms of mir-138 were predicted by two programs. miR-138-5p was proposed as a potential obesity biomarker, since its expression in serum was significantly higher in obese subjects [17]; miR-138-5p was downregulated during adipogenic differentiation [125]. Only TargetScan predicted the binding of miR-27b-3p, which was reported as an important factor in the regulation of adipogenesis [126] The expression of miR-27b-3p was up-regulated in mice with a high-fed diet [127], but it decreased during the adipogenesis of human multipotent adipose-derived stem cells. The overexpression of miR-27b-3p during adipogenesis blunted the induction of peroxisome proliferator-activated receptors (PPAR) gamma and C/EBPα, two key adipogenesis regulators, as well as repressing triglyceride accumulation [128]. MiR-27b-3p was also shown to negatively regulate adipogenesis by impairing mitochondrial biogenesis, structure, and activity [129]. Moreover, miR-27b-3p was demonstrated to play a central role in the pathogenesis of glucocorticoid-induced central fat accumulation. The overexpression of miR-27b-3p had similar effect as dexamethasone treatment on inhibition of brown adipose differentiation [130]. miR-27-3p directly regulates lysyl oxidase and contributes to repressing adipogenic lineage commitment [131]; furthermore, miR-27-3p inhibits adipocyte differentiation via the suppression of cyclic adenosine monophosphate (cAMP) response element binding protein expression [132].

### 2.8. GPx8

The last member of the GPx family, GPx8, is the non-Sec-containing monomeric enzyme. Generally, a great deal of information about GPx8 has yet to be determined. GPx8 occurs mainly as an intrinsic membrane peroxidase of the endoplasmic reticulum (ER), with its active site protruding into the ER lumen. GPx8 uses protein disulfide isomerase as a reductant more efficiently than GSH and together with GPx7 is involved in protein folding. Ramming et al. confirmed the role of GPx8 in the prevention of H_2_O_2_ leakage from ER [133], with the involvement of GPx8 in calcium homeostasis and signaling reported recently [134]. GPx8 is transcriptionally regulated by hypoxia-inducible factor 1-α, with GPx8 depletion affecting insulin signaling cascades [135]. GPx8 is regarded as a lung-abundant enzyme, since it is decreased during influenza pneumonia and is stabilized as lung tissue starts to regenerate [5,135]. It is also considered one of the cellular substrates of the hepatitis C virus [136].

*GPx8* has an extra-long 3′UTR, the TargetScan predicted more than 1600 unique miRNAs if poorly conserved sites are also included (setting used for other GPxs). Therefore only conserved miRNAs were included in this analysis to reduce the number of explored miRNAs. Some miRNAs predicted to regulate GPx8 were mentioned above, e.g., miR-185-5p or miR-133a-3p. Other interesting miRNAs in relation to obesity include miR-26b-5p, which is upregulated in mature adipocytes and may play a role in the development of obesity [137]. miR-26b-5p regulates the proliferation of human preadipocytes via the arrest of the G1/S transition partly by targeting cyclin D2 [138]. The expression of miR-486-5p was upregulated in the plasma of obese children [139]. Furthermore, the overexpression of miR-486-5p induces a premature senescence-like phenotype and directly regulates sirtuin 1, a major regulator of longevity and metabolic disorders [140]. miR-223-3p had been reported to be abnormally expressed in several inflammation diseases and may well have an important role as the biomarker and a therapeutic target of these disorders [141]. Obese and overweight subjects displayed a lower level of circulating miR-223-3p, which increased significantly three months after lifestyle intervention [142].

## 3. Summary

miRNAs are important players in post-transcriptional regulation. To repress the expression of target mRNA, miRNAs recognize target sites, leading to mRNA degradation or translation inhibition. Most of the target mRNAs studied to date are regulated through miRNA interactions with their 3′UTRs, therefore we focused our analysis only on this region despite increasing evidence of 5′UTR and coding sequences targeting as well as noncanonical miRNA regulation. The flexibility in miRNA-targeting rules makes it difficult to predict reliable miRNAs, thus the use of more than one predicting program is common practice. Here, four programs were used with different algorithms, with intersections within the predictions sought after (Figure 2). Furthermore, we used miRWalk as a source of validated miRNA-GPxs interaction in order to supplement the results.

Nowadays, it is known that miRNA regulate most of the human genes. We focused on the family of glutathione peroxidases, and indeed confirmed that all GPxs were predicted to be a target of many miRNAs (Table 1 and Table S1). The number of unique miRNas correlates well with the 3′UTR length of each individual *GPxs* (Figure 1) [143]. As previously reported, shorter 3′UTR are more common for housekeeping genes [144]. *GPx1* and *GPx4* have the shortest 3′UTR, followed by *GPx2*, with these genes representing the most common GPxs. On the other hand, *GPx8* has the longest 3′UTR, which suggests that this gene might possess an as-of-yet overlooked spatially or temporally distinct function, a role which can be tightly regulated by various miRNAs.

GPxs represent important antioxidant enzymes in mammals and these miRNA have been implicated in many pathways from proliferation and apoptosis to the modulation of many diseases connected to oxidative stress and inflammation. Intriguingly, as inflammation has been shown to have a distinct role in obesity, the involvement of GPxs variants in obesity has been reported repeatedly. The oxidative stress responsive miRNAs play a role in altering the expression of antioxidant genes that quench ROS to maintain redox homeostasis. Furthermore, the intracellular redox balance during adipocyte differentiation is tightly regulated by ROS-reducing enzymes, including GPxs, with GPx1, -3 and -4 representing major selenoproteins abundantly expressed in white adipose tissue [145]. A recent thorough review by Brandao et al. summarizes the involvement of miRNAs in adipocyte formation and obesity [146]. The intersection of identified miRNAs as putative GPxs regulators and obesity studies is summarized in Table 2. Several of the mentioned miRNAs could possibly regulate more than one GPx. One interesting miRNA predicted to regulate four GPxs is let-7i-5p, which has been reported to play a crucial role in the modulation of function of brite (brown-to-white) adipocytes, the thermogenic fat cells in white adipose tissue [147]. miR-24-3p, predicted for three GPxs, plays a complex role in adipogenesis and mature adipocytes. This miRNA promotes adipocyte differentiation by direct targeting MAPK7 signaling [69]. On the other hand, Kang et al. reported the inhibitory effect of miR-24 on adipocyte differentiation and maturity through targeting the fatty acid-binding protein 4 [148].

Five GPxs represent selenoproteins that contain specific amino acid Sec in their sequence. Sec is the essential part of the catalytic site in Sec-GPxs (1–4, 6), one that enables reducing hydroxyperoxide at the expense of glutathione [149]. The synthesis of selenoproteins follows an unusual mechanism, since Sec is encoded by an in-frame UGA stop codon [150]. Sec incorporation requires complex machinery involving a specific class of tRNA^Sec^ binding proteins and translation initiation factors, all of which are highly regulated by selenium availability. Selenium deficiency leads to alterations in the pathways controlling inflammatory, endoplasmic reticulum unfolded protein response, and protein biosynthetic processes. A specific secondary structure present in the 3′UTR of selenoprotein mRNAs named SECIS (selenocystein insertion sequence) is necessary for selenoprotein specific UGA recoding and Sec insertion. Since miRNA regulation also depends on the accessibility of a target site, one would expect that the competing secondary structure within 3′UTR could prevent the binding of a miRNA induced silencing complex [151]. On the contrary, Maciel-Dominguez has proposed the possibility that the regulation of selenoproteins by miRNA might also take place through the modulation of Sec incorporation [38]. Indeed, the regulatory effect of miRNAs on selenoproteins GPx1 and GPx2 was reported as experimentally proved [21,22].

Single miRNA can recognize target sequences of other gene transcripts. Genes within one biochemical pathway or a network could conceivably be jointly co-regulated by a common miRNA. A typical example is miR-181a, which is involved in thymic development at multiple stages [152]. Moreover, the miRNA regulation of molecular pathways may be implicated in disease phenotype [153]. One example of this is miR-200, which functions as a multifunctional tumor suppressor in meningiomas [154]. Therefore, the possibility was explored that other GPxs are regulated by a specific miRNA, and we found more than a hundred unique miRNAs possibly targeting three or more GPxs (Table S2). Two miRNAs, miR-30c-3p and miR-185-5p, appear to be the most interesting. A proadipogenic miRNA, miR-30c-5p [155] was predicted to solely regulate GPx3, whereas miR-30c-3p, a miRNA from the other arm of the precursor, was predicted to be a regulator of five other GPxs (Table S2). This is quite intriguing, as this miRNA could possibly having an important yet undescribed function in the antioxidant system. miR-185-5p was identified by several programs to regulate six of eight GPxs. As this miRNA was altered by selenium deficiency, it seems to be a master regulator and can play a role in feedback control of the expression of selenoproteins [38].

Interestingly both mature miRNAs from pre-mir-125a were reported in connection to obesity. Three GPxs were predicted to be regulated by miR-125a-3p, which was upregulated in the adipose tissue of obese subjects [156]. miR-125a-3p promotes adipogenesis via the suppression of RhoA/ROCK1/ERK1/2 pathway [157], with circulating levels responding to a fatty acid enriched diet [48].

Among redoximiRs, miR-34a-5p and miR-34c-5p were predicted to regulate three GPxs. In terms of obesity, miR-34a-5p seems to be extremely important, as a significantly increased expression of miR-34a-5p has been observed in the serum and subcutaneous adipose of obese diabetic subjects [158]. A study by Fu et al. [159] identified miR-34a-5p as a potential target for treating obesity-related diseases due to inhibition of beige and brown fat formation [159]. An increased level of miR-34a in diabetic mice resulted in induced β-cell apoptosis [160]. Recent studies revealed that aberrantly elevated miR-34a-5p in obese subjects directly targeted β-Klotho, the obligate co-receptor for the metabolic hormones fibroblast growth factor-19 (FGF19) and FGF21, and in this way attenuated their signaling in energy metabolism [161]. Conversely, when wild-type (WT) and miR-34a(−/−) mice were fed a high-fat diet for 24 weeks, the miR-34a(−/−) mice grew significantly heavier, with a greater increase in white adipose tissue weight than was the case with WT [158].

## 4. Conclusions

In our thorough exploration of the possible miRNAs that interact with *GPxs* 3′UTRs our results showed that expression of all GPxs might in fact be regulated by many miRNAs. A high number of miRNAs was predicted to regulate two or more GPxs. Some of these show a clear association with obesity and related pathologies. We identified the most interesting candidates that are worth exploring further to clarify the conjunction of GPxs, miRNAs, and obesity. These findings might contribute to the understanding of the mechanisms of the development of obesity-related pathologies.

## Figures and Tables

**Table 1:** In silico prediction of miRNAs targeting 3′UTR of *GPxs* employing four different algorithms.

Gene	Ensembl Gene ID	3′UTR Length (nt)	TargetScan ^a^	miRDB ^a^	miRanda ^a^	DIANA ^a^
*GPx1*	ENSG00000233276.2	222	99	16	25	37
*GPx2*	ENSG00000176153	336	133	10	38	62
*GPx3*	ENSG00000211445.7	863	440	57	90	54
*GPx4*	ENSG00000167468.12	263	124	11	10	3
*GPx5*	ENSG00000224586.2	785	328	35	84	46
*GPx6*	ENSG00000198704	996	414	41	101	68
*GPx7*	ENST00000361314.4	626	236	28	100	57
*GPx8*	ENSG00000164294.9	4869	15	46	53	111

^a^ Total number of unique mature miRNAs targeting a selected gene predicted by each individual software application.

**Table 2:** List of identified miRNAs as putative regulators of respective GPxs with reported obesity association.

Gene	Identified miRNA	Observed Effect *	Note	Reference
*Gpx1*	miR-376a-3p	↓obese serum		[17]
	miR-367b-3p	↓peripheral blood mononuclear cells	non-responders in weight-loss trial	[25]
*Gpx2*	miR-17-5p	↑obese subjects		[39]
	miR-27a-5p	↑serum of obese children		[53]
*Gpx3*	miR-146b-5p	↑adipose tissue in mice	high fat diet	[1]
		↓in monocytes of obese persons		[61]
	miR-379-5p	↑adipose tissue in mice	high fat diet	[1]
	miR-575	expressed in the amnion of obese woman only		[66]
	miR-642a-3p	↑in fat depots of obese subjects		[67]
	miR-4269	↓in obese donors		[68]
	miR-24-3p	↑liver high fat diet mice		[70]
*Gpx4*	miR-324-3p	↓gestational obesity	associated with pregnancy weight gain	[76]
	miR-1908-5p	involvement in regulation of obesity development		[80]
*Gpx5*	miR-143-3p	↓in obese human subject		[86]
	miR-194-5p	↓liver - High fat maternal diet altered		[90]
*Gpx6*	miR-146a/b-5p	↑adipose tissue in mice	high fat diet	[1]
		↓in monocytes of obese persons		[61]
	miR-9	↑adipose tissue in obese pigs		[95]
*Gpx7*	miR-29 family	obesity related		[105]
	let-7 family	obesity related		[112]
	mir-133b-3p	↓ adipose tissue in mice	high fat diet	[1]
	miR-204-5p	↑ adipose tissue in mice	high fat diet	[1]
	miR-335-5p	↑adipose tissue in mice	genetically fat mice/high fat diet	[118, 119]
	miR-138-5p	↑serum of obese subjects	potential biomarker of obesity	[17]
	miR-27b-3p	↑atria in mice	high fat diet	[127]
*GPx8*	miR-486-5p	↑plasma of obese children		[139]
	miR-223-3p	↓in serum obese subjects	after life style intervention	[142]

* MicroRNAs downregulated (↓) or upregulated (↑).

## Supplementary Materials

The following are available online at http://www.mdpi.com/1422-0067/19/4/1199/s1.

## Abbreviations

3′UTR	3′untranslated region
5′UTR	5′untranslated region
A/U	adenin/uracil
AMP	adenosine monophosphate
ApoE	apolipoprotein E
BMI	body mass index
cAMP	cyclic adenosine monophosphate
C/EBP	CCAAT-enhancer-binding protein
cGPx	cytosolic glutathione peroxidase
CLASH	crosslinking, ligation, and sequencing of hybrids
ER	endoplasmic reticulum
FGF19	fibroblast growth factor 19
GI	gastrointestinal
GSH	glutathione
GPx	glutathione peroxidase
HUVEC	human umbilical vein endothelial cells
LDL	low density lipoprotein
MAPK7	mitogen-activated protein kinase 7
mGPx	mitochondrial glutathione peroxidase
miRNA	microRNA
MnSOD	manganese superoxide dismutase
NFκB	nuclear factor-κB
NPHGPx	nonselenocysteine containing phospholipid hydroperoxide glutathione peroxidase
Nrf2	nuclear factor erythroid 2-related factor 2
PHGPx	phospholipid hydroperoxide glutathione peroxidase
PKA1	protein kinase A1
PKA/C/EBPβ	protein kinase A/CAAT box/enhancer-binding protein beta
PPAR	peroxisome proliferator-activated receptor
ROS	reactive oxygen species
Se	selenocystein insertion sequence
SECIS	serine
snGPx	sperm nuclear glutathione peroxidase
SNP	single nucleotide polymorphism
SOD	superoxide dismutase
T2DM	type 2 diabetes mellitus
TNFα	tumor necrosis factor alpha
TrxR2	thioredoxin reductase 2
Wnt	wngless/integration-1
WT	wild-type
