# Supplementary material for: MicroRNAs as Potential Regulators of Glutathione Peroxidases Expression and Their Role in Obesity and Related Pathologies
Source: Int J Mol Sci. 2018 Apr 14;19(4):1199. doi: 10.3390/ijms19041199 (PMC5979329; doi:10.3390/ijms19041199)
Supplement: Supplementary file 1 [file ijms-19-01199-s001.zip › Supplementary Materials.docx]

**Supplementary Materials: MicroRNAs as Potential Regulators of Glutathione Peroxidases Expression and Their Role in Obesity and Related Pathologies**

**Supplement 1:** Details to methods-Bioinformatic analysis

**Supplement 2:** Table S1—Overview of mature miRNAs targeting selected genes predicted by each individual software.

**Supplement 3:** Table S2—Overview of number of software that predicted each mature miRNA targeting each GPX.

**Supplement 1**

**Bioinformatic analysis**

Since different miRBase releases are implemented in different programs, to compare our results we adopted the 3p/5p nomenclature according to current miRBase release 21. We made an effort to correctly interconnect all the additional specific miRNA reports mentioned with this nomenclature. Nevertheless, we sometimes had to rely on a higher number of sequence reads based on deep sequencing annotation in miRBase, as no further identification (sequence, accession ID, -3p, -5p or -*) was provided in the text.

TargetScan

The **TargetScanHuman** (http://www.targetscan.org/vert_71/) algorithm is a statistical model that predicts the effects of miRNAs binding to canonical sites. The model takes into account 14 different features (e.g. miRNA site, number of sites, mRNA site context—site conservation, including the mRNA sequence around the site, AU content, secondary structure) which are combined to from the context++ score of the miRNA targeting efficacy [1]. The current version of this algorithm uses mature miRNA sequences downloaded from miRBase release 21 [2]. For our analysis, we included broadly conserved, conserved, and poorly conserved groups of miRNAs.

DIANA-microT

**DIANA-microT-CDS**, the 5th version of the microT algorithm (http://diana.imis.athena-innovation.gr/DianaTools/), is specifically trained on a positive and a negative set of MREs located in both the 3'-untranslated region (UTR) and coding sequences (CDS) [3]. The algorithm identifies the optimal alignment between the miRNA extended seed sequence and calculates the conservation score based on the evolutionary conservation of a MRE in 16 species. The current version of this algorithm uses mature miRNA sequences downloaded from miRBase release 18, which is compatible with 3p/5p nomenclature.

miRanda

**MicroRNA.org** (http://www.microrna.org/microrna/getGeneForm.do) is a web-accessible information resource for miRNA target predictions and expression profiles. The target sites are predicted using the **miRanda** algorithm, with the likelihood of mRNA downregulation **mirSVR** used for scoring. The calculation of mirSVR uses a regression model that is trained on sequence and contextual features of the predicted miRNA::mRNA duplex; this includes the duplex features, A/U composition near the target sites as well as secondary structure accessibility and global features such as length of 3´UTR or conservation score. Mature miRNA sequences were downloaded from miRBase release 15.0. For our analysis, we selected all miRNAs with “good” mirSVR scores.

miRDB

**miRDB** (http://www.mirdb.org/miRDB/index.html) is a database that contains miRNA target predictions performed with the **MirTarget V3** algorithm, a process was developed by analyzing
high-throughput expression profiling data in a support vector machine framework. Unlike most other prediction algorithms, MirTarget predicts both conserved and nonconserved gene targets by treating target site conservation as an important but non-required sequence feature [4]. Currently, miRDB hosts mature miRNAs derived from miRBase release 21. For our analysis, we used a default target score of 50 or above as the confidence level for potentially functional miRNA targets.

miRWalk

**miRWalk2.0** (http://zmf.umm.uni-heidelberg.de/apps/zmf/mirwalk2/index.html) is a database of predicted and experimentally verified miRNA-target interactions that enables the comparison of putative binding sites from various programs [5]. We used this program only to search for validated miRNA-target interaction in order to supplement the results we obtained from the above mentioned four prediction programs and to analyze possible intersections.

**References**

1. Agarwal, V.; Bell, G. W.; Nam, J. W.; Bartel, D. P. Predicting effective microRNA target sites in mammalian mRNAs. *Elife* **2015,** *4*.
2. Kozomara, A.; Griffiths-Jones, S. miRBase: Annotating high confidence microRNAs using deep sequencing data. *Nucleic Acids Res.* **2014,** *42*, D68–D73.
3. Paraskevopoulou, M. D.; Georgakilas, G.; Kostoulas, N.; Vlachos, I. S.; Vergoulis, T.; Reczko, M.; Filippidis, C.; Dalamagas, T.; Hatzigeorgiou, A. G. DIANA-microT web server v5.0: Service integration into miRNA functional analysis workflows. *Nucleic* *Acids Res.* **2013**, *41*, W169-W173.
4. Wong, N.; Wang, X. W. miRDB: An online resource for microRNA target prediction and functional annotations. *Nucleic Acids Res.* **2015**, *43*, D146-D152.
5. Dweep, H.; Gretz, N. miRWalk2.0: A comprehensive atlas of microRNA-target interactions. *Nat. Methods* **2015**, *12*, 697.
